# Genetic background of adaptation of Crimean-Congo haemorrhagic fever virus to the different tick hosts

**DOI:** 10.1371/journal.pone.0302224

**Published:** 2024-04-25

**Authors:** Seyma S. Celina, Jiří Černý

**Affiliations:** Faculty of Tropical AgriSciences, Center for Infectious Animal Diseases, Czech University of Life Sciences Prague, Prague, Czech Republic; Lerner Research Institute - Cleveland Clinic, UNITED STATES

## Abstract

Crimean-Congo haemorrhagic fever orthonairovirus (CCHFV) is a negative-sense, single-stranded RNA virus with a segmented genome and the causative agent of a severe Crimean-Congo haemorrhagic fever (CCHF) disease. The virus is transmitted mainly by tick species in *Hyalomma* genus but other ticks such as representatives of genera *Dermacentor* and *Rhipicephalus* may also be involved in virus life cycle. To improve our understanding of CCHFV adaptation to its tick species, we compared nucleotide composition and codon usage patterns among the all CCHFV strains i) which sequences and other metadata as locality of collection and date of isolation are available in GenBank and ii) which were isolated from in-field collected tick species. These criteria fulfilled 70 sequences (24 coding for S, 23 for M, and 23 for L segment) of virus isolates originating from different representatives of *Hyalomma* and *Rhipicephalus* genera. Phylogenetic analyses confirmed that *Hyalomma-* and *Rhipicephalus-*originating CCHFV isolates belong to phylogenetically distinct CCHFV clades. Analyses of nucleotide composition among the *Hyalomma-* and *Rhipicephalus-*originating CCHFV isolates also showed significant differences, mainly in nucleotides located at the 3^rd^ codon positions indicating changes in codon usage among these lineages. Analyses of codon adaptation index (CAI), effective number of codons (ENC), and other codon usage statistics revealed significant differences between *Hyalomma*- and *Rhipicephalus*-isolated CCHFV strains. Despite both sets of strains displayed a higher adaptation to use codons that are preferred by *Hyalomma* ticks than *Rhipicephalus* ticks, there were distinct codon usage preferences observed between the two tick species. These findings suggest that over the course of its long co-evolution with tick vectors, CCHFV has optimized its codon usage to efficiently utilize translational resources of *Hyalomma* species.

## 1. Introduction

The Crimean-Congo haemorrhagic fever orthonairovirus (CCHFV), recently renamed as *Orthonairovirus haemorrhagiae* by the International Committee on Taxonomy of Viruses (ICTV) Nairoviridae Study Group, is a member of the genus *Orthonairovirus*, family *Nairoviridae* and the etiologic agent of Crimean-Congo haemorrhagic fever (CCHF) disease [1]. It is a lipid-enveloped, single-stranded, negative-sense RNA virus with a segmented genome. Each of the three CCHFV genomic segments has a different function: S (small) segment encodes nucleocapsid protein (N), M (medium) segment the glycoproteins (Gn and Gc), and L (large) segment RNA-dependent RNA polymerases (RdRp) [2].

CCHF is the most widely distributed tick-borne viral disease in humans being endemic in many geographic regions across Africa, Asia, Eastern and Southern Europe, and the Middle East [2]. In Africa and Eurasia, CCHFV is among the deadliest human pathogens [3] and outbreaks of CCHF pose a significant threat due to its epidemic potential, high case fatality rates ranging from 5% to 80% [4], potential for nosocomial outbreaks, and challenges in treatment and prevention [5]. Therefore, due to its high potential for human-to-human transmission, the high risk of laboratory-acquired infections, and the lack of a specific vaccine, CCHFV is classified as a biosafety level 4 (BSL-4) agent [6].

The virus is transmitted through the bite of its main vector, ticks in the genus *Hyalomma* (Ixodidae). *Hyalomma marginatum* Koch, 1844 is the major vector of CCHFV in the Old World [2]. Additionally, various other species within the *Hyalomma* genus have also been reported to carry the CCHFV, such as *H*. *excavatum* Koch, 1844, *H*. *lusitanicum* Koch, 1844, *H*. *rufipes* Koch, 1844, and *H*. *truncatum* Koch, 1844 [7–10], playing significant roles as vectors in the Middle East, the Iberian Peninsula, and Africa, respectively [11]. However, the scope of potential vectors expands beyond *Hyalomma* species, with over 30 different tick species implicated in CCHFV transmission [11]. Ticks from other genera of Ixodidae, including *Rhipicephalus* and *Dermacento*r, are also capable of transmitting CCHFV. *Rhipicephalus* ticks, such as *R*. *bursa* Koch‎, 1844‎, and *R*. *turanicus* Pomerantsev, 1936 have been identified as carriers of CCHFV in regions spanning Albania, Turkey, Greece, and Russia [12–16]. Similarly, in genus *Dermacentor*, *D*. *marginatus* Sulzer, 1776 has tested positive for CCHFV in Turkey, Russia, and Spain [12,14,15,17,18], highlighting its potential as a vector for the virus. Despite the detection of CCHFV within *D*. *marginatus* eggs and its confirmed competency as a tick vector for the virus in laboratory studies, questions remain regarding their natural vectorial capacity within the enzootic cycle of the virus [11,19].

Ticks transmit CCHFV to various mammals by taking blood meals for their maturation and egg production. Nevertheless, humans can acquire CCHFV infection not only from tick bites but also from direct contact with the blood or tissues of infected animals or human patients. Other possible routes of transmission for CCHFV are through infected mother to offspring, sexual contact, and blood transfusion [20].

Due to its complex ecology, CCHFV is characterized by a great genetic diversity with complex evolutionary patterns [21]. CCHFV can be phylogenetically divided into eight distinguishable clades (Africa 1–3, Europe 1–3, and Asia 1 and 2). These clades differ not only by their geographic distribution and primary sequence but also by other features as pathogenic potentials. On the other hand, segment reassortment between the clades and long-range migration events of individual CCHFV lineages were observed, demonstrating the dynamic nature of CCHFV evolution [22,23].

In the genomes of each species, there is a distinct preference for specific synonymous codons, which encode the same amino acids, leading to unequal frequencies of codon usage within their respective genes [24,25]. This concept, referring to the differential usage of synonymous codons, is known as codon usage bias. Codon usage bias has been studied in a wide range of organisms, from prokaryotes to eukaryotes and viruses. However, how such biases arise is a much-debated area of molecular evolution. Different factors have been suggested to be related to codon usage bias, including gene expression level, guanine-cytosine content, guanine-cytosine skew, amino acid conservation, protein hydropathy, and transcriptional selection [26–29].

The codon usage bias in RNA viruses is mainly affected by compositional constraints under mutational pressure and natural selection [30,31]. Many studies on codon usage bias in various viruses have shown that the main driver shaping codon usage patterns is mutational pressure than natural selection [32–34]. However, for many DNA and RNA viruses, mutational pressure is not the only factor on establishing codon usage patterns [35,36]. Compared with prokaryotic and eukaryotic genomes, the viral genome has certain features, such as dependence on its hosts for replication, protein synthesis, and transmission of proteins. The interaction between virus and host is considered to affect survival, adaptation and evolution of virus, as well as its ability to evade the host’s immune system. In many viruses, including CCHFV, an optimal codon usage pattern has been shown to be an important factor in better adaptation of these viruses to their hosts [37,38]. Moreover, major ecological shifts in the evolution of viruses have been linked to variations in their codon usage [39]. On the other hand, codon usage pattern deoptimization leads to decrease of fitness in many viruses [40–42].

Previously, an analysis describing the adaptation of CCHFV codon usage pattern to its vertebrate hosts was performed [36], but the tick species (except for *Hyalomma* ticks) were not included in the study, despite the fact that arthropod vectors have at least the same effect on arbovirus evolution as their vertebrate hosts [43].

Therefore, we performed a comprehensive analysis of codon usage patterns of three genomic segments (S, M, and L) of CCHFV isolates from *Hyalomma* and *Rhipicephalus* (no sequences of CCHFV strains isolated from other tick species are available) using available sequence data. While *Hyalomma* species are the primary virus reservoirs and vectors for CCHFV, there is experimental evidence suggesting the potential involvement of ticks from the *Rhipicephalus* genus in its transmission; however, concrete evidence supporting their significant role in viral maintenance and natural transmission remains inconclusive [19]. Recent studies have detected CCHFV antigen within a *Rhipicephalus* species, along with viral RNA in different *Rhipicephalus* species across Albania, Kosovo, Greece, Turkey, and Iran [12–16,44–46]. Notably, this highlights the exclusive presence of CCHFV strains belonging to Europe 2 (clade VI) within *R*. *bursa* species, indicating their potential role as vectors of the virus [13]. Conversely, *R*. *sanguineus* sensu lato ticks are commonly linked with the Europe 1 clade. Further laboratory investigations are essential to establish the vector competence of *Rhipicephalus* species [13]. Moreover, a recent comprehensive review categorizes *R*. *bursa* among confirmed vectors for CCHFV [11]. This classification is based on documented infection rates, records, and observations spanning over 30 distinct tick species [11]. Given this context, our study aimed to investigate the codon usage patterns of CCHFV in relation to both *Hyalomma* and *Rhipicephalus* tick species, providing a deeper understanding of its adaptation to various vector species.

## 2. Materials and methods

### 2.1 Data collection

In this study, complete or nearly complete genome sequences of CCHFV strains isolated from tick species were analyzed, while partial sequences, that could lead to biased results in terms of codon usage, were excluded. We compared nucleotide composition and codon usage patterns among the all CCHFV strains i) which sequences and other metadata as locality of collection and date of isolation are available in GenBank and ii) which were isolated from in-field collected tick species. These criteria fulfilled 70 sequences (24 coding for S, 23 for M, and 23 for L segment) of CCHFV isolates originating from different representatives of *Hyalomma* and *Rhipicephalus* genera, and were retrieved from the National Center for Biotechnology Information (NCBI) GenBank database (https://ncbi.nlm.nih.gov/nuccore/) on December 2019, together with information about isolation date, collection locality, and tick species. Sequence details were compiled in **S1 Table.**

### 2.2 Phylogenetic analysis

Sequences were divided into groups according to CCHFV segments and aligned using MAFFT (v7.427). Maximum clade credibility (MCC) trees were constructed using Bayesian evolutionary analysis by sampling trees (BEAST, version 1.10.4) [47,48] using HKY as the nucleotide substitution model with gamma distributed rate heterogeneity and a relaxed molecular clock. The Markov chain Monte Carlo (MCMC) algorithm was executed for 100-million generations, with the initial 10% of the chain discarded as burn-in using the TreeAnnotator package integrated within the BEAST software [47]. The resulting MCC tree files were subsequently imported into the Figtree tool (http://tree.bio.ed.ac.uk/software/figtree/) for tree visualization and to estimate the time to the most recent common ancestor (tMRCA). Clades with Bayesian posterior probabilities exceeding 0.5 were displayed in the trees. A total of 70 sequences were used in this analysis.

### 2.3 Nucleotide composition analysis

Nucleotide compositional properties of the CCHFV coding sequences were calculated using CAIcal server (http://genomes.urv.es/CAIcal/) [49]. The overall frequency of occurrence of nucleotides (A%, C%, U% and G%), frequency of each nucleotide at the third site of synonymous codons (A3%, C3%, U3% and G3%), frequencies of occurrence of nucleotides GC at the first (GC1), second (GC2) and third synonymous codon positions (GC3), the mean frequencies of nucleotide GC at the first and the second position (GC12), overall GC and AU contents, and AU and GC contents at the third codon positions (AU3, GC3) were calculated. AUG and UGG that are only the codons for Met and Trp (no synonymous codons) along with the termination codons (UAG, UAA, and UGA) which do not encode any amino acids were excluded from the analyses since they were not expected to show any codon usage bias.

### 2.4 Analysis of the effective number of codons (ENC)

An effective number of codons (ENC) analysis was used to assess codon usage bias in CCHFV segments isolated from *Hyalomma* and *Rhipicephalus* ticks, calculated with CodonW software (v1.4.4) (http://sourceforge.net/projects/codonw). ENC values typically range from 20 to 61, with lower values signifying extreme codon usage bias and higher values indicating the opposite.

To explore whether the codon usage of given strains is solely due to mutational pressure or selection pressure, an ENC-plot was produced. To determine the correlation between the expected ENC and the GC3s values, the expected ENC values were calculated for different GC3s using the method of Singh et al. (2016) [50]:

$ENCexp=2+s+\frac{29}{\left(s^{2}+{\left(1-s\right)}^{2}\right)}$ where “s” indicates GC contents at the 3rd synonymous codon positions (GC3s%). When data points align with or near the standard curve, it suggests predominantly mutational pressure, while points falling below indicate codon usage subject to natural selection.

### 2.5 Relative Synonymous Codon Usage (RSCU) analysis

RSCU analysis was performed to compare the codon usage preferences of three different segments of CCHFV being isolated from *Hyalomma* or *Rhipicephalus*. RSCU was calculated using CAIcal server. RSCU values greater than 1 show positive codon usage bias and are described as “abundant” codons. The values less than 1 show negative codon usage bias and are described as “less-abundant” codons. A RSCU value of 1 indicates no bias in codon usage.

*Hyalomma*- and *Rhipicephalus*-isolated CCHFV coding sequences were compared with codon usage values of their natural vectors obtained from Codon and Codon Pair Usage Tables (CoCoPUTs) (https://hive.biochemistry.gwu.edu/review/codon2) [51], accessed in June 2020, and analyzed using CAIcal server.

### 2.6 Codon adaptation index (CAI) analysis

CAI was performed to gain insight into the codon preferences of CCHFV in relation to its tick species. The CAI values vary from 0 to 1, and higher values indicate higher levels of codon usage bias towards the codons used in highly expressed genes [40]. The most frequent codons signify the highest relative adaptation to the host, and sequences having higher CAI are known to be favoured over sequences having lower CAI. The CAI analysis was performed using CAIcal server for *Hyalomma* (*H*. *anatolicum*, *H*. *asiaticum*, *H*. *dromedarii*, *H*. *excavatum*, *H*. *lusitanicum*, *H*. *marginatum*, *H*. *rufipes* and *H*. *truncatum*) and *Rhipicephalus* (*R*. *bursa* and *R*. *sanguineus*) species. The reference data sets showing codon frequencies for these tick species were obtained from the CoCoPUTs database.

In addition, the expected CAI values (eCAI) at the 95% confidence interval were calculated in order to ascertain whether statistically significant differences in CAI values result from codon preferences, and to provide statistical support to CAI analyses, a Kolmogorov-Smirnov test for the eCAI was also applied [52].

### 2.7 Correspondence analysis (COA)

COA analysis was performed to detect the variation of codon usage data [58]. The COA was performed based on RSCU values for CCHFV strains (S, M, and L segments) isolated from *Hyalomma* and *Rhipicephalus*, and the distribution of the strains in the plane of the first two principal axes of COA was determined. CodonW (v1.4.4) software was used in order to examine the codon usage indices.

### 2.8 Selection analysis

The dN/dS ratio (ω) is used to compare the non-synonymous substitution rate per non-synonymous site (dN) with the synonymous substitution rate per synonymous site (dS). This ratio estimates the selective pressures acting on a coding sequence by identifying the fraction of codons that are evolving under purifying/negative selection (ω < 1), nearly neutral evolution (ω = 1), or positive/diversifying selection (ω > 1). To estimate the selection pressure on the S, M, and L segments of CCHFV isolated from *Hyalomma* and *Rhipicephalus* ticks, the Hypothesis Testing Using Phylogenies software v2.2.4 (HyPhy) was used [53]. Pervasive site-specific selection pressure analysis was performed in this study, utilizing Fast Unconstrained Bayesian AppRoximation (FUBAR) method accessible at the Datamonkey webserver [54] (https://www.datamonkey.org/). The dN/dS estimates for selection inference were analyzed using the posterior probability of ≥0.9.

### 2.9 Statistical analysis

A statistically significant difference between all nucleotide compositional properties of *Hyalomma-* and *Rhipicephalus*-isolated CCHFV strains (A, C, U, G, A3, U3, G3, C3, AU, GC, GC1, GC2, AU3, GC3, GC12), and among ENC and CAI values was addressed by applying the t-test and Wilcoxon & Mann-Whitney test with Bonferroni correction (p < 0.05) in GraphPad Prism 9.

## 3. Results

### 3.1 *Hyalomma* and *Rhipicephalus* originating CCHFV isolates are phylogenetically isolated

**Fig 1:**
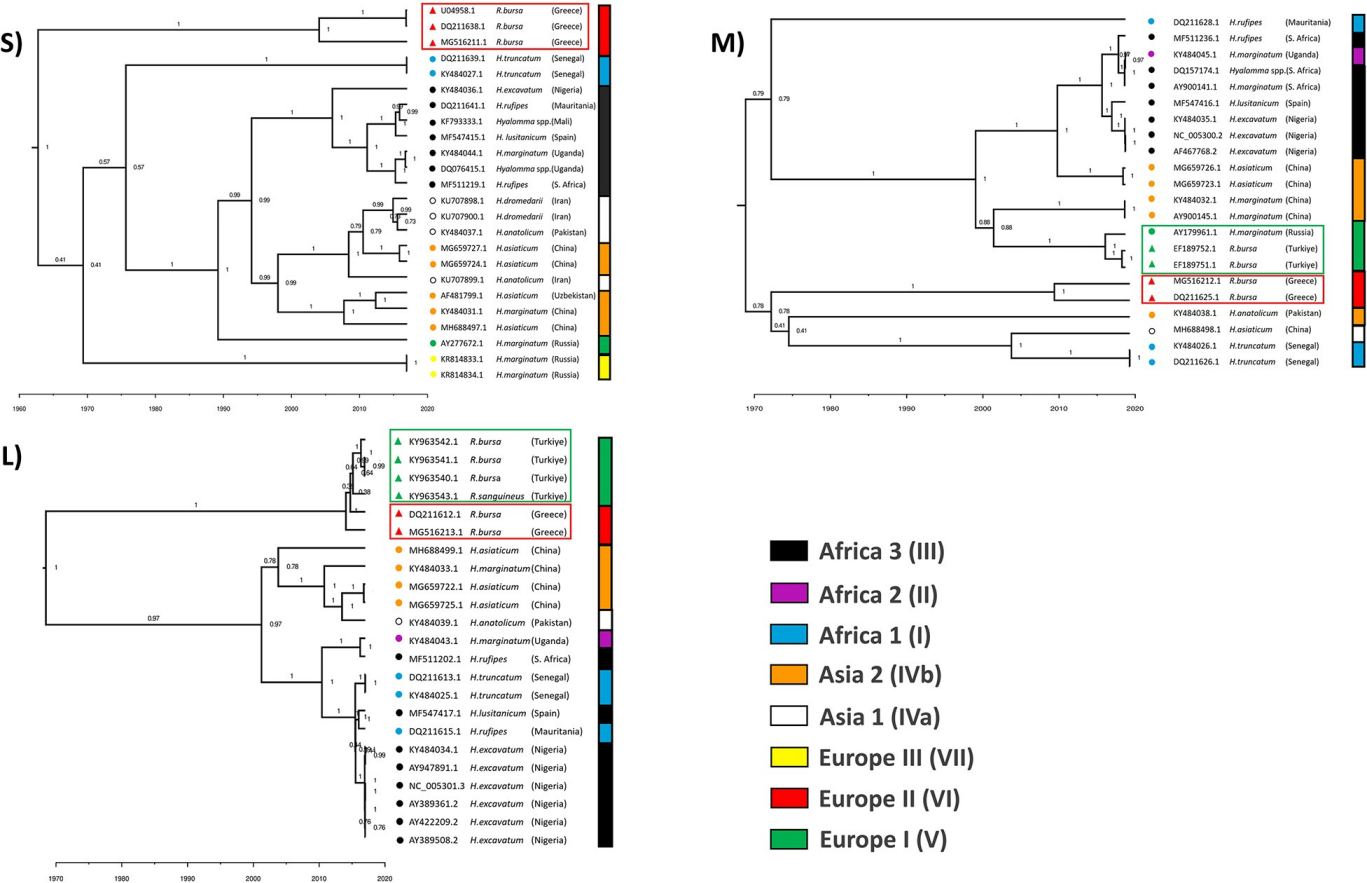
The maximum clade credibility tree for 70 CCHFV strains isolated from *Hyalomma* and *Rhipicephalus* species. The tree node shows the tMRCA in years. *Hyalomma*- and *Rhipicephalus*-isolated strains are distinguished in branches of different shapes (circle for *Hyalomma*-isolated strains and triangle for *Rhipicephalus*-isolated strains). Phylogenetic clades of strains are represented in the legend with different colors.

The phylogenetic trees of the S and L segments showed the genomic sequences were clearly assembled into clusters in relation to their tick hosts (**Fig 1**). The results revealed that CCHFV strains isolated from *Rhipicephalus* were genetically distant from *Hyalomma*-isolated strains. This highlighted the evidence of strong selection pressure on host adaptation.

In contrast, the phylogenetic tree for the M segment did not exhibit a clear separation of *Hyalomma*- and *Rhipicephalus*-isolated CCHFV strains. Notably, CCHFV strains isolated from *R*. *bursa* in Greece (Europe 2) formed a distinct cluster from *Hyalomma* strains, while strains from *R*. *bursa* in Turkey (Europe 1) clustered together with *Hyalomma*-isolated strains. Previous studies showed that codon usage patterns in viral genomes can be influenced by geographic origins [36,50,55–58]. Therefore, the clustering of M segment strains may reflect both host adaptation and geographic distribution.

### 3.2. Nucleotide composition of CCHFV isolates from *Hyalomma* and *Rhipicephalus* ticks is different

**Fig 2:**
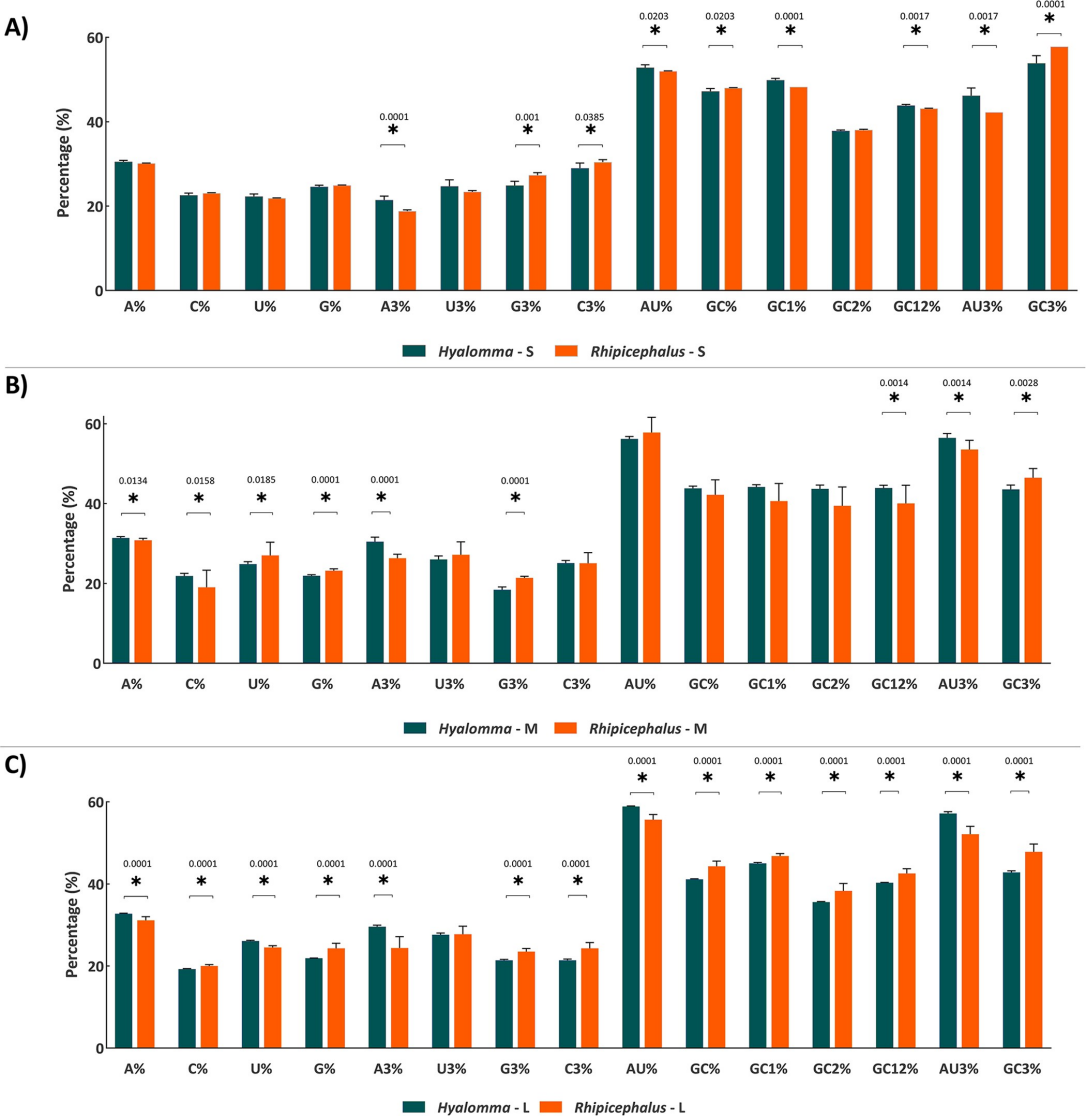
Nucleotide content distribution and composition in *Hyalomma*- and *Rhipicephalus*-isolated S (A), M (B), and L (C) segments, respectively. Standard deviation is marked in the plot by the error bars. Asterisk (*) shows a significant difference between variables (p < 0.05).

Nucleotide composition strongly affects codon usage patterns. Important are especially nucleotides at the third position of codons (A3, U3, G3, C3) [37,39,66]. There were statistically significant differences between *Hyalomma*- and *Rhipicephalus*-isolated CCHFV strains in the frequency of A3 and G3 in all segments (**Fig 2; S2 Table**).

Further, C3 was significantly different between *Hyalomma*- and *Rhipicephalus*-isolated CCHFV strains in S and L (but not M) segments (**Fig 2; S2 Table**). No statistically significant difference was observed for U3 in any genomic segment of CCHFV strains isolated from *Hyalomma* and *Rhipicephalus*.

Moreover, GC content at all positions of codons (GC1, GC2, and GC3) and GC content at the first plus the second positions of codons (GC12) also play an important role in influencing overall codon usage preferences [32,59]. In S and L segments, GC content significantly differed between *Hyalomma*- and *Rhipicephalus*-isolated CCHFV strains on almost all codon positions (**Fig 2; S2 Table**). In M segment, GC content significantly differs between *Hyalomma*- and *Rhipicephalus*-isolated CCHFV strains only at GC3 and GC12 positions. Nevertheless, results of these analyses showed substantial differences in frequencies of occurrence of nucleotides between *Hyalomma*- and *Rhipicephalus*-isolated CCHFV variants (p < 0.05).

### 3.3 *Hyalomma*- and *Rhipicephalus*-isolated CCHFV strains preferentially use different codons

**Fig 3:**
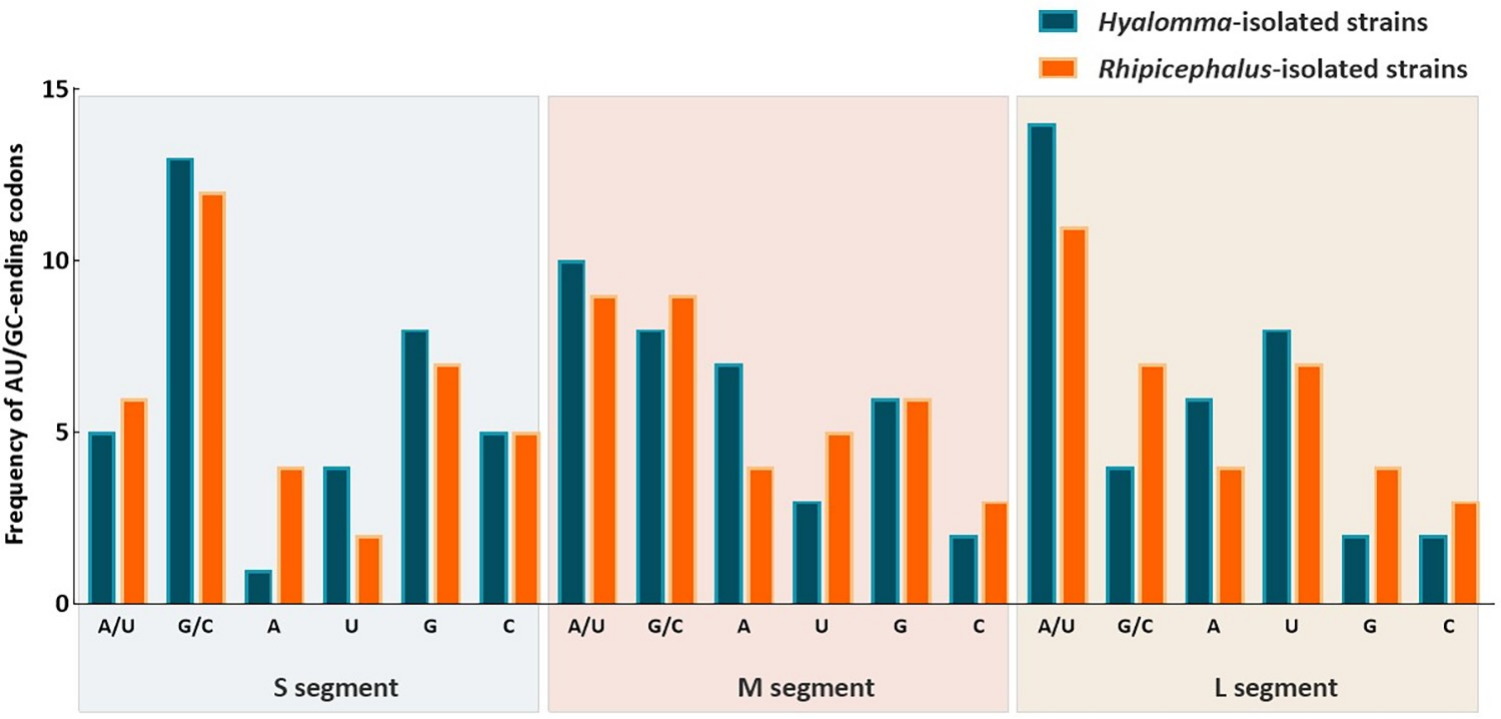
Preference for A/U- and G/C-ending codons, as well as A-, U-, G-, and C-ending codons separately, among *Hyalomma*- and *Rhipicephalus*- isolated S, M and L segments.

At S segments, G/C-ended codons were preferred over A/U-ended codons in CCHFV strains isolated from both *Hyalomma* and *Rhipicephalus* tick species. But, *Rhipicephalus*-isolated CCHFV strains had a strong preference to the A-ended codons whereas *Hyalomma*-isloated ones had a higher frequency in U-ended codons (**Fig 3**, **Table 1**).

**Table 1:** The relative synonymous codon usage frequency (RSCU) of *Hyalomma*- and *Rhipicephalus*-isolated CCHFV strains.

AA	CODONS	CCHFV—S SEGMENT			CCHFV—M SEGMENT			CCHFV—L SEGMENT		
		*Hyalomma*	*Rhipicephalus*	Difference	*Hyalomma*	*Rhipicephalus*	Difference	*Hyalomma*	*Rhipicephalus*	Difference
Phe	UUU	0.96	0.85	0.11	1.09	1.33	0.24	1.14	1.16	0.02
	UUC	1.04	1.15	0.11	0.91	0.68	0.23	0.86	0.84	0.02
Leu	UUA	0.13	0	0.13	0.98	0.83	0.15	0.83	0.28	0.55
	UUG	0.56	0.84	0.28	1.17	1.19	0.02	1.32	**1.76**	0.44
	CUU	**2.13**	**1.80**	0.33	1.08	1.49	0.41	1.10	1.15	0.05
	CUC	1.43	1.57	0.14	0.67	0.62	0.05	0.92	1.22	0.3
	CUA	0.44	0.33	0.11	1.05	0.81	0.24	0.93	0.71	0.22
	CUG	1.30	1.46	0.16	1.06	1.08	0.02	0.91	0.89	0.02
Ile	AUU	1.25	1.20	0.05	1.13	1.41	0.28	1.20	**1.64**	0.44
	AUC	0.99	1.23	0.24	0.84	0.62	0.22	0.68	0.60	0.08
	AUA	0.76	0.57	0.19	1.03	0.98	0.05	1.12	0.76	0.36
Val	GUU	1.03	0.89	0.14	1.28	1.26	0.02	1.28	1.34	0.06
	GUC	1.23	1.18	0.05	0.96	1.18	0.22	0.86	0.80	0.06
	GUA	0.27	0.49	0.22	0.72	0.29	0.43	0.62	0.58	0.04
	GUG	1.47	1.43	0.04	1.04	1.27	0.22	1.23	1.28	0.05
Ser	UCU	1.46	1.49	0.03	0.83	0.47	0.36	1.18	0.88	0.3
	UCC	1.08	1.05	0.03	0.58	0.48	0.1	0.61	0.67	0.06
	UCA	0.97	0.72	0.25	**1.74**	1.05	0.69	1.15	0.50	0.65
	UCG	0.26	0.22	0.04	0.18	0.16	0.02	0.20	0.08	0.12
	AGU	1.02	0.72	0.3	1.21	**1.61**	0.4	1.40	**1.74**	0.34
	AGC	1.21	**1.82**	0.61	1.46	**2.23**	0.77	1.47	**2.13**	0.66
Pro	CCU	1.17	1.19	0.02	1.25	1.43	0.18	1.42	0.57	0.85
	CCC	0.49	0.77	0.28	0.83	0.41	0.42	0.76	0.90	0.14
	CCA	**1.85**	**1.68**	0.17	**1.71**	**1.93**	0.22	1.52	**2.44**	0.92
	CCG	0.49	0.35	0.14	0.22	0.24	0.02	0.29	0.09	0.2
Thr	ACU	0.98	1.16	0.18	1.0	0.66	0.34	1.21	0.86	0.35
	ACC	1.58	1.16	0.42	0.91	0.96	0.05	1.14	**1.88**	0.74
	ACA	1.31	1.30	0.01	**1.78**	**2.00**	0.22	1.44	0.98	0.46
	ACG	0.13	0.37	0.24	0.30	0.39	0.09	0.21	0.29	0.08
Ala	GCU	1.02	0.85	0.17	0.97	0.58	0.39	1.10	0.92	0.18
	GCC	1.36	1.39	0.07	1.13	**1.92**	0.79	0.62	0.46	0.16
	GCA	1.46	**1.63**	0.27	**1.78**	1.44	0.34	**2.02**	**2.19**	0.17
	GCG	0.15	0.13	0.02	0.12	0.06	0.06	0.26	0.42	0.16
Tyr	UAU	0.62	0.72	0.1	0.83	0.87	0.04	0.99	0.59	0.4
	UAC	1.38	1.28	0.1	1.17	1.13	0.04	1.01	1.41	0.4
His	CAU	0.71	0.56	0.15	0.93	0.53	0.4	1.23	**1.64**	0.41
	CAC	1.29	1.44	0.15	1.07	1.47	0.4	0.77	0.36	0.41
Glu	CAA	0.70	0.36	0.34	0.86	0.60	0.26	0.95	0.55	0.4
	CAG	1.30	**1.64**	0.34	1.14	1.40	0.26	1.05	1.45	0.4
Asn	AAU	0.64	0.70	0.06	0.97	1.12	0.15	1.01	1.11	0.1
	AAC	1.36	1.30	0.06	1.03	0.88	0.15	0.99	0.90	0.09
Lys	AAA	0.85	0.65	0.2	1.12	1.02	0.1	1.13	1.25	0.12
	AAG	1.15	1.35	0.2	0.88	0.99	0.11	0.87	0.75	0.12
Asp	GAU	0.93	0.67	0.26	0.97	1.12	0.15	1.13	1.47	0.34
	GAC	1.07	1.33	0.26	1.03	0.88	0.15	0.87	0.54	0.33
Glu	GAA	0.84	0.59	0.25	1.24	0.99	0.25	1.27	1.55	0.28
	GAG	1.16	1.41	0.25	0.76	1.01	0.25	0.73	0.45	0.28
Cys	UGU	1.25	1.33	0.08	0.98	0.84	0.14	1.07	0.93	0.14
	UGC	0.75	0.67	0.08	1.02	1.16	0.14	0.93	1.08	0.15
Arg	CGU	1.34	0.47	0.87	0.13	0.06	0.07	0.19	0.09	0.1
	CGC	0.23	0.59	0.36	0.16	0.04	0.12	0.29	0.53	0.24
	CGA	0.16	0.35	0.19	0.14	0.04	0.1	0.33	0.11	0.22
	CGG	0.37	0.12	0.25	0.14	0.06	0.08	0.21	0.03	0.18
	AGA	**1.71**	**1.76**	0.05	**3.49**	**3.89**	0.4	**2.66**	**2.04**	0.62
	AGG	**2.20**	**2.70**	0.5	**1.93**	**1.90**	0.03	**2.32**	**3.21**	0.89
Gly	GGU	0.77	1.14	0.37	1.00	0.73	0.27	1.26	1.38	0.12
	GGC	1.26	1.03	0.23	1.23	1.55	0.32	0.91	1.16	0.25
	GGA	1.20	1.22	0.02	1.06	1.10	0.04	1.14	0.80	0.34
	GGG	0.77	0.61	0.16	0.72	0.62	0.1	0.69	0.68	0.01

AA represents amino acid; grey colour represents the most optimal codons favoured by CCHFV isolated from two different tick hosts (the highest RSCU value for each particular amino acid); the bold represents over-represented codons (RSCU ≥ 1.6), the underline represents under-represented codons (RSCU ≤ 0.6) codons. Green color indicates the highest difference between RSCU values of *Hyalomma*- and *Rhipicephalus*-isolated CCHFV for each segment, while the red color shows the lowest difference.

At M segments, A/U-ended codons were highly preferred in CCHFV strains isolated from both *Hyalomma* and *Rhipicephalus* tick species. M segments of *Hyalomma-*isolated CCHFV strains showed a higher preference to the A-ended codons, while M segments of CCHFV strains isolated from *Rhipicephalus* had a preference to highly use U-ended codons. Regarding the G/C-ended codons, M segments of CCHFV strains isolated from both tick species used different codons, but both preferred to use rather C-ended than G-ended codons (**Fig 3**, **Table 1**).

At L segments, CCHFV strains isolated from both tick species also had a high preference for A/U-ended codons. *Hyalomma-*isolated strains prefer A-ending codons, while *Rhipicephalus*-isolated strains prefer U-ending codons. However, L segments of *Rhipicephalus*-isolated CCHFV strains had higher frequencies in C-ended codons over G-ended codons (**Fig 3**, **Table 1**).

**Fig 4:**
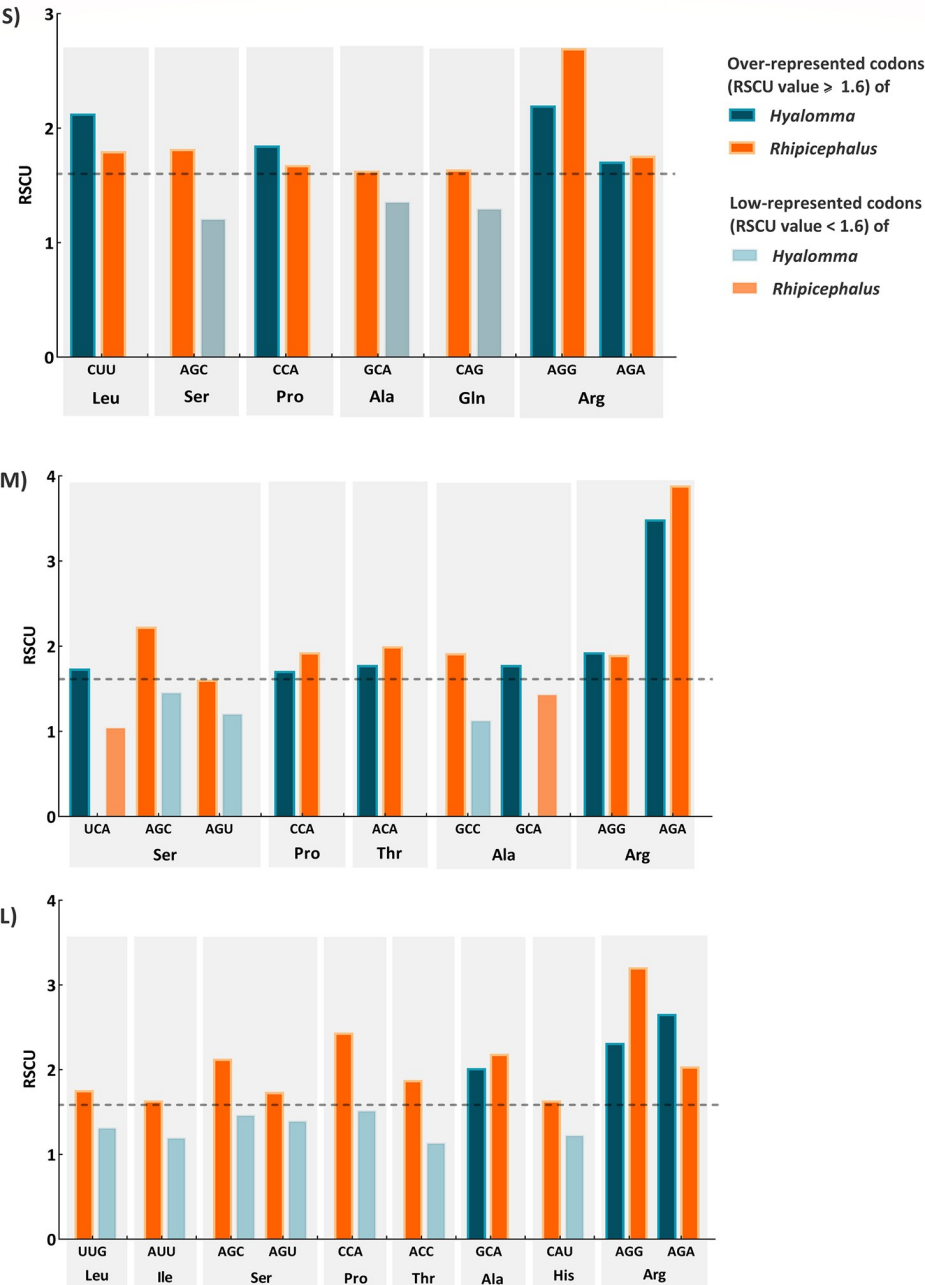
Over-represented (RSCU ≥ 1.6) and low-represented codons (RSCU < 1.6) between *Hyalomma*- and *Rhipicephalus*- isolated CCHFV strains for S, M and L segments.

Codon over- and underrepresentation analysis emphasized that RSCU values of the majority codons ranged from 0.6 to 1.6. Interestingly, we found that the nucleotide frequencies at the end of most over-represented codons (RSCU > 1.6) differ between *Hyalomma*- and *Rhipicephalus*-isolated CCHFV strains (**Fig 4**). Pro, Ala, and Arg are over-represented in all *Hyalomma*- and *Rhipicephalus* isolated CCHFV genomic segments. However, *Hyalomma*-isolated strains have a strong preference on GCA for Ala, while *Rhipicephalus* ones prefer to use GCC. Moreover, *Hyalomma*- and *Rhipicephalus* isolated L segments differ on codon usage preferences for Arg, where AGA is over-represented in *Hyalomma*-isolated strains, and *Rhipicephalus* ones show a higher tendency to use AGG codon (**Fig 4**).

**Fig 5:**
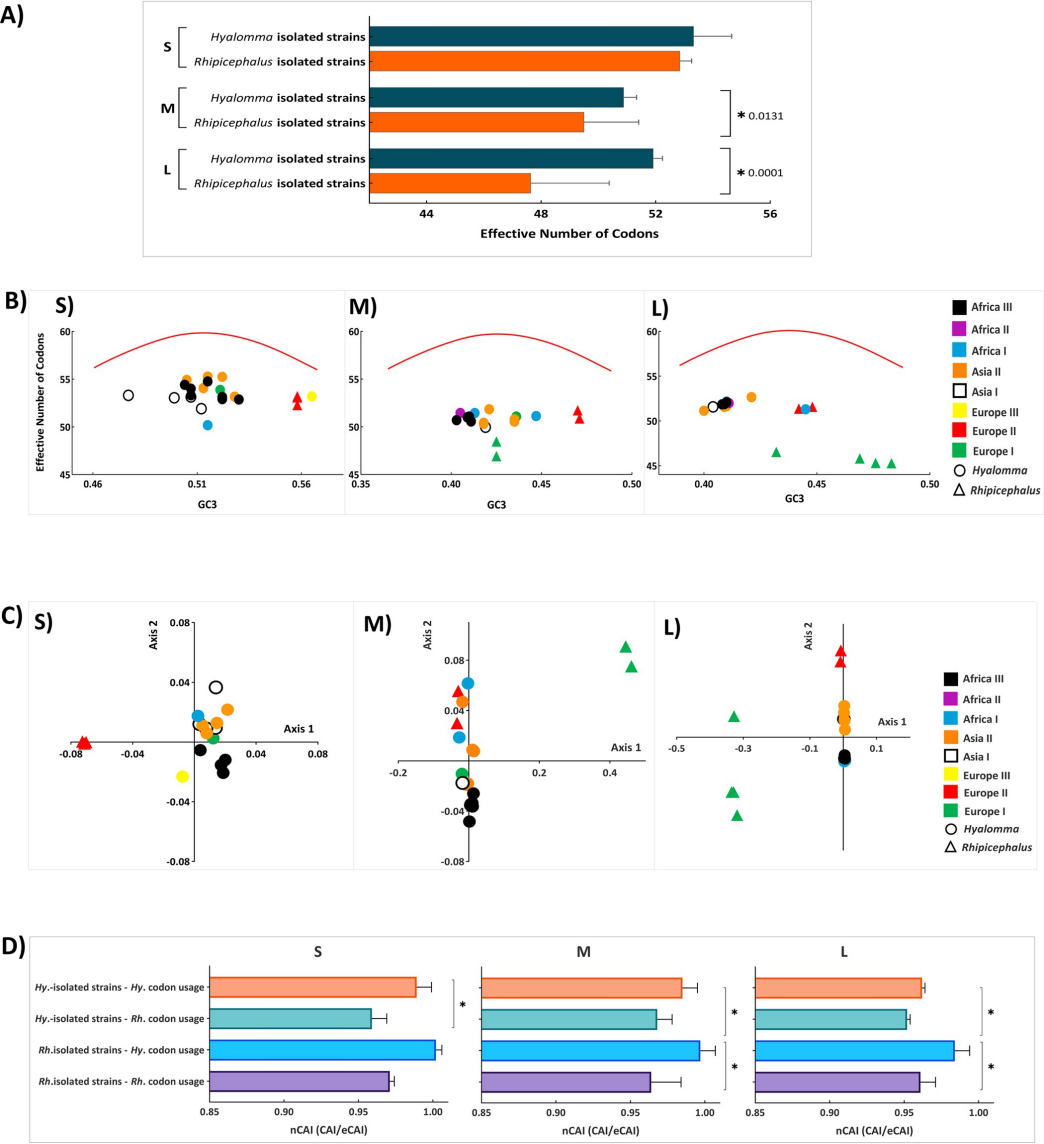
Effective number of codons (ENC), correspondence analysis (COA) and codon adaptation index (CAI) analyses of *Hyalomma*- and *Rhipicephalus*-isolated CCHFV strains. **A)** Comparison of the values for ENC between *Hyalomma*- and *Rhipicephalus*-isolated CCHFV strains for S, M, and L segments. Standard deviation is marked in the plot by the error bars. **B)** ENC-GC3 plots of *Hyalomma*- and *Rhipicephalus*-isolated CCHFV genomes. ENC values (Y-axis) was plotted against the GC content at the third synonymous codon positions (GC3s values, X-axis). The curve (red line) indicates the expected codon usage if GC compositional constraints alone account for the codon usage bias. *Hyalomma*- and *Rhipicephalus*-isolated CCHFV strains are marked by different symbols (circle for *Hyalomma*-isolated strains and triangle for *Rhipicephalus*-isolated strains). Different colors mark for different CCHFV phylogenetic clades. **C)** COA values are based on the RSCU values of *Hyalomma*- and *Rhipicephalus*-isolated S, M, and L segments, respectively. **D)** nCAI values of *Hyalomma*-isolated strains to *Hyalomma* and *Rhipicephalus* codon usage, and *Rhipicephalus*-isolated strains to *Hyalomma* and *Rhipicephalus* codon usage in relation to S, M, and L segments, respectively.

Analysis of ENC values alone showed significant differences between the *Hyalomma*- and *Rhipicephalus*- isolated CCHFV strains for M and L but not for S segments (p < 0.05) (**Fig 5A**). While the S segment exhibited relatively similar ENC values for both *Hyalomma* (53.33 ± 1.33) and *Rhipicephalus* (52.85 ± 0.41), the M segment had higher ENC values for *Hyalomma*-isolated strains (50.89 ± 0.45) compared to *Rhipicephalus*-isolated strains (49.50 ± 1.91), suggesting a difference in codon usage bias among CCHFV isolates from two tick hosts. Further, the L segment showed the greatest difference in ENC values, with *Hyalomma*-isolated strains (51.92 ± 0.32) having markedly higher values than *Rhipicephalus*-isolated strains (47.64 ± 2.74), indicating a significant variation in codon usage bias of the L segment of CCHFV strains isolated from both hosts. When ENC was plotted as a function of GC3s (GC content at the third synonymous codon position), we could see a weak but significant codon usage bias for both *Hyalomma*- and *Rhipicephalus*-isolated CCHFV strains (**Fig 5B**). Further, it was apparent that both *Hyalomma*- and *Rhipicephalus*- isolated CCHFV strains form separated clusters. The results of COA revealed that CCHFV strains isolated from *Hyalomma* and *Rhipicephalus* ticks are separated into distinct clusters based on codons used by these strains (**Fig 5C**). This separation cannot be explained based on affiliation to the CCHFV phylogenetic clades. It clearly shows that *Hyalomma*- and *Rhipicephalus*-isolated CCHFV strains have considerable variation in codon usage patterns.

### 3.4 Both *Hyalomma*- and *Rhipicephalus*-isolated CCHFV strains show higher codon adaptation index values for *Hyalomma* tick species

All genomic segments of both *Hyalomma*- and *Rhipicephalus*-isolated CCHFV strains show significantly higher codon adaptation index values for *Hyalomma* than for *Rhipicephalus* tick species (p < 0.05). Interesting is the fact that M and L segments of *Rhipicephalus*-isolated CCHFV strains show significantly higher CAI values to *Hyalomma* ticks than the M and L segments of *Hyalomma*-isolated CCHFV strains (p = 0.0001) (**Fig 5D**).

### 3.5 The pattern of selection in *Hyalomma*- and *Rhipicephalus*-isolated CCHFV strains varies

We utilized the FUBAR method, a site-specific pervasive selection approach, to assess the selective pressure on 70 CCHFV sequences isolated from both *Hyalomma* and *Rhipicephalus* ticks. The estimated dN/dS ratios were as follows: 0.04 and 0.02 for S segment, 0.05 and 0.17 for M segment, and 0.04 and 0.07 for L segment isolated from *Hyalomma* and *Rhipicephalus*, respectively. The results further revealed that the *Hyalomma*- and *Rhipicephalus*-isolated S segments had 380 and 103 sites identified under negative/purifying selection, respectively. For the *Hyalomma*- and *Rhipicephalus*-isolated M segments, 1696 and 397 sites were identified under negative/purifying selection, respectively. However, no sites were identified under positive/diversifying selection in both segments isolated from two ticks. Lastly, on the *Hyalomma*- and *Rhipicephalus*-isolated L segments, 2858 and 334 sites were identified under negative/purifying selection, respectively. Additionally, 1 site was identified under positive/diversifying selection for *Rhipicephalus*-isolated L segments.

Overall, these findings suggest that there are variations in the selective pressures acting on CCHFV isolates of different tick hosts, which may be influenced by the segment-specific codon usage biases and the tick species from which they were isolated.

## 4. Discussion

The differences in genome composition and codon usage patterns between *Hyalomma*- and *Rhipicephalus*-isolated CCHFV variants can influence viral fitness, evolution, and the ability to replicate within different tick species as well as mammalian hosts.

Phylogenies of S and L genomic segments of *Hyalomma*- and *Rhipicephalus*-isolated CCHFV variants revealed a clear phylogenetic separation of *Rhipicephalus*-isolated CCHFV strains from *Hyalomma* ones. These findings diverge from previous phylogenetic analyses, which primarily emphasized the influence of geographical spots on codon usage patterns [36]. Instead, our study highlights the evidence of strong selection pressure on host adaptation, which is in agreement with CAI analysis. Our findings suggest that, beyond geographical factors, vector host species may significantly impact the codon usage patterns of the virus. Interestingly, the results of the phylogenetic analysis showed a phylogenetic separation among Europe 2 and Europe 1 clades in terms of the ticks in which the strains are vectored. Europe 1 circulates in various geographic regions, including southern Russia, Turkey, the Balkan Peninsula, and Iran. The strains belong to Europe 1 (clade V) are known to be highly pathogenic in humans and *Hyalomma* species were observed to harbor particularly this clade. Likewise, the infection caused by strains belonging to Europe 2 (clade VI) has mild or non-pathogenic effects on humans, and this clade is exclusively found in ticks of the *Rhipicephalus* genus, in particular *R*. *bursa* [13,60]. In contrast to the clear separation observed in the phylogenetic trees of the S and L segments based on hosts, the M segment tree did not reveal a distinct clustering of *Hyalomma*- and *Rhipicephalus*-isolated CCHFV strains. Previous studies showed that geographic origins might influence the codon usage patterns of the viral genome [36,50,55–58], and our findings suggest that a combination of both host adaptation and geographical origin may have contributed to the observed patterns in codon usage bias of *Hyalomma*- and *Rhipicephalus*-isolated CCHFV M segments. Additionally, potential variations in two different ticks hosting the same genetic structure of the virus should be explored, utilizing artificial feeding experiments to facilitate such investigations, thereby offering valuable suggestions for further studies in this field.

Previously, it has been shown that codon usage bias, or the preference for one type of codon over another, can be greatly influenced by the overall nucleotide composition in the genome [32,59]. The nucleotide composition analysis revealed substantial differences on frequencies of occurrence of nucleotides between *Hyalomma*- and *Rhipicephalus*-isolated CCHFV variants. Despite the differences, it is apparent that the M and L segments of *Hyalomma*- and *Rhipicephalus*-isolated CCHFV strains are AU- rich; and A/U-ended codons appear to be preferred, indicating that the usage of optional codons might be influenced by compositional constraints resulting in the presence of mutational pressure. This is consistent with previous report indicating a substantial portion of mammalian-host isolated CCHFV strains are enriched with AU [36]. Further, this result is similar to the other RNA viruses such as West Nile virus [61], dengue virus [62], Marburg virus [59], Ebola virus [63] and bluetongue virus [57] where A/U-ended codons appear to be preferred. However, S segments isolated from both tick species are more GC rich and preferentially use G/C-ended codons. The biological importance of this condition is uncertain; therefore, it is important to investigate the factors influencing different nucleotide frequencies of CCHFV segments [64].

Previous studies on codon usage bias have also suggested that the composition of amino acids is a key factor in determining the nucleotide contents at the first and second codon positions of viral genomes, while the variation in proteins was forced by functional selection. However, at the third codon positions of a viral gene, a large proportion of the possible alterations (69%) result in synonymous or silent mutations that are not constrained by the functional selection of amino acids [64]. Based on RSCU values, we explored the different codon usage preferences of *Hyalomma*- and *Rhipicephalus*-isolated CCHFV sequences. In the previous study, it was observed that *Hyalomma*-isolated CCHFV strains exhibited a preference for C-ended codons [36]. However, our study revealed that only CCHFV S segments isolated from *Hyalomma* and *Rhipicephalus* ticks demonstrated a preference for C-ended codons, while M and L segments show contrary preferences toward A- and U-ended codons. Remarkably, *Hyalomma*-isolated M segments have a higher tendency to use A-ended codons, while strains isolated from *Rhipicephalus* have a strong preference to use U-ended codons. For L segment, although the two hosts have similar preferences for having more A-ended codons over U, they exhibit different codon preferences for the same amino acids such as Arg, Cys, and Thr. Furthermore, the previous study noted that CCHFV strains isolated from *Hyalomma* ticks exhibited a preference for CGC codons for Arg, while AGA and AGG were less favored [36]. Conversely, our analysis revealed an over-representation of AGA and AGG codons across S, M, and L segments of CCHFV isolates from *Hyalomma* and *Rhipicephalus* ticks. Regarding the codon preference for Ala, the previous study reported a strong preference for GCC in *Hyalomma*-isolated strains [36]. However, our findings showed that GCA was the preferred codon for Ala in *Hyalomma*-isolated S, M, and L segments. Additionally, our study revealed a strong preference for CCA codons for Pro in both *Hyalomma*- and *Rhipicephalus*-isolated CCHFV strains. This contrasts with the findings of the previous study [36], which indicated a preference for CCC codons in *Hyalomma*-isolated CCHFV strains.

All genomic segments of *Hyalomma*- and *Rhipicephalus*-isolated CCHFV strains have remarkably different codon usage patterns. The codon usage bias of CCHFV isolated from two tick genera was found to be low, with ENC values higher than 35. Similar low codon usage bias has also been reported among several other RNA viruses for instance, Zika virus (ENC: 53.32) [58], Ebola virus (ENC: 57.23) [65], chikungunya virus (ENC: 55.56) [35], classical swine fever virus (ENC: 51.7) [66], foot-and-mouth virus (ENC: 51.53) [67], hepatitis C virus (ENC: 52.62) [68], Venezuelan equine encephalitis virus (ENC: 56.51) [56], and West Nile virus (ENC: 53.81) [61]. It has been indicated that the low codon usage bias of the virus is beneficial for the efficient replication in its host cells and the reduced competition between virus and its hosts for protein synthesis. Similarly, all segments of tick-isolated CCHFV strains show rather high ENC values which indicate low codon usage bias. It suggests that the evolution of low codon bias within CCHFV coding sequences has allowed it to successfully maintain its survival cycle within its tick vectors as well as mammalian hosts.

The influence of a tick vector on CCHFV codon usage pattern was also visible from CAI analysis. Comparative analysis of CAI values revealed that both *Hyalomma*- and *Rhipicephalus*-isolated CCHFV strains display higher adaptation to use the codons that are preferred by *Hyalomma* tick species. These results suggest that over the course of its long co-evolution with tick vectors, CCHFV has optimized its codon usage patterns to utilize the translational resources of *Hyalomma* species more efficiently than that of *Rhipicephalus* ticks which are vectors used only by specific CCHFV. Higher genetic adaptation of CCHFV strains isolated from the tick species of two genera, favoring the use of codons preferred by *Hyalomma* ticks, can be attributed to the role of *Hyalomma* species as the primary vectors of CCHFV. Contrarily, a lower adaptation of *Hyalomma*- and *Rhipicephalus*-isolated CCHFV segments to *Rhipicephalus* codon usage highlights that *Rhipicephalus* ticks are rather occasional vectors or evolutionary new vectors that are used by CCHFV in areas where *Hyalomma* ticks are either absent or at least rare.

The selection analysis of *Hyalomma*- and *Rhipicephalus*- isolated S, M, and L segments suggest that CCHFV isolates from these hosts are subject to strong purifying (negative) selection, as has been previously observed for other RNA viruses such as Zika virus, the West Nile virus, the dengue virus, the yellow fever virus, and the tick-borne encephalitis virus, which exhibit low dN/dS ratios ranging from 0.019 to 0.066 [69].

Overall, the results of this study show the strong difference in codon usage patterns between the CCHFV strains isolated from different tick species, which may mirror the differences in evolutionary processes that forms these virus strains. However, our study has identified a few limitations that should be considered when interpreting our findings. Firstly, the sample size of 70 sequences (24 for S segment, 23 for M segment, and 23 for L segment) of CCHFV isolates from different representatives of the *Hyalomma* and *Rhipicephalus* genera may not be sufficient to provide robust conclusions. Additionally, there are limitations in the amount of full CCHFV genome sequences isolated from *Rhipicephalus* and the lack of CCHFV sequences from *Dermacentor* species, despite experimental evidence suggesting their potential involvement in virus transmission and the frequent detection of viral RNA in these species [11–16,18,19,44–46]. Thus, generating full genome sequences of CCHFV strains isolated from *Dermacentor* ticks could provide valuable insights into the virus’s adaptation to additional tick species. These limitations highlight the importance of generating full genome sequences of CCHFV strains from their tick hosts to achieve a more thorough understanding of the virus-vector coevolution. Despite a few limitations, this research not only provided knowledge about the variation in CCHFV codon usage patterns in relation to their two vectors but also contributed to analyzing the factors that influence the adaptation of the virus to its vector species. In silico studies are highly important in the case of CCHFV as it is regarded to be a BSL-4 pathogen, and therefore, studies on the virus are very limited.

## 5. Conclusion

Our study suggests that analysis of codon usage bias of *Hyalomma*- and *Rhipicephalus*-isolated CCHFV strains can provide an alternative strategy to understand the evolution and genetic background of adaptation of CCHFV to its vector species. Our findings indicate that CCHFV strains isolated from *Hyalomma* and *Rhipicephalus* have significant differences in codon usage variations and patterns. Furthermore, our study highlighted that *Hyalomma*- and *Rhipicephalus*-isolated CCHFV strains have a higher tendency to use the codons that are preferred by species of *Hyalomma* genus. The results of this study indicate the strong effect of evolutionary processes on codon usage patterns and highlight the evidence of strong selection pressure on host adaptation while codon usage bias patterns in *Hyalomma*- and *Rhipicephalus*-isolated CCHFV M segments may result from a combination of host adaptation and geographical origin. The data analyzed in this study contribute to our understanding of the virus’s evolution and genetic adaptation to its vector species.

## Supporting information

S1 Table The strain name, accession number, origin, isolation host and collection date of polyprotein-coding region of each CCHFV isolates used in this study. (XLSX)

S2 Table Nucleotide composition analysis of S, M, and L segments of CCHFV isolated from *Hyalomma* and *Rhipicephalus* species (%). Significant values on the 95% confidence limit between strains isolated from two tick hosts are marked in bold (p<0.05).
(XLSX)
